# Identification of Amino Acid Residues Responsible for Inhibition of Host Gene Expression by Influenza A H9N2 NS1 Targeting of CPSF30

**DOI:** 10.3389/fmicb.2018.02546

**Published:** 2018-10-24

**Authors:** Laura Rodriguez, Aitor Nogales, Munir Iqbal, Daniel R. Perez, Luis Martinez-Sobrido

**Affiliations:** ^1^Department of Microbiology and Immunology, University of Rochester, Rochester, NY, United States; ^2^Agencia Española de Medicamentos y Productos Sanitarios, Madrid, Spain; ^3^Avian Viral Diseases Programme, The Pirbright Institute, Woking, United Kingdom; ^4^Department of Population Health, Poultry Diagnostic and Research Center, University of Georgia, Athens, GA, United States

**Keywords:** influenza, H9N2, NS1, interferon, host gene expression, virus–host interactions, mammalian adaptation, virus evolution

## Abstract

H9N2 influenza A viruses (IAV) are considered low pathogenic avian influenza viruses (LPAIV). These viruses are endemic in poultry in many countries in Asia, the Middle East and parts of Africa. Several cases of H9N2-associated infections in humans as well as in pigs have led the World Health Organization (WHO) to include these viruses among those with pandemic potential. To date, the processes and mechanisms associated with H9N2 IAV adaptation to mammals are poorly understood. The non-structural protein 1 (NS1) from IAV is a virulence factor that counteracts the innate immune responses. Here, we evaluated the ability of the NS1 protein from A/quail/Hong Kong/G1/97 (HK/97) H9N2 to inhibit host immune responses. We found that HK/97 NS1 protein counteracted interferon (IFN) responses but was not able to inhibit host gene expression in human or avian cells. In contrast, the NS1 protein from earlier H9N2 IAV strains, including the first H9N2 A/turkey/Wisconsin/1/1966 (WI/66), were able to inhibit both IFN and host gene expression. Using chimeric constructs between WI/66 and HK/97 NS1 proteins, we identified the region and amino acid residues involved in inhibition of host gene expression. Amino acid substitutions L103F, I106M, P114S, G125D and N139D in HK/97 NS1 resulted in binding to the 30-kDa subunit of the cleavage and polyadenylation specificity factor (CPSF30) and, in consequence, inhibition of host gene expression. Notably, changes in the same amino acid residues resulted in the lack of inhibition of host gene expression by WI/66 NS1. Importantly, our results identified a new combination of amino acids required for NS1 binding to CPSF30 and inhibition of host gene expression. These results also confirm previous studies demonstrating strain specific differences in the ability of NS1 proteins to inhibit host gene expression.

## Introduction

Influenza A viruses (IAV) are members of the *Orthomyxoviridae* family and are classified in subtypes based on the antigenic features of the two surface glycoproteins present on the viral envelop: hemagglutinin (HA) and neuraminidase (NA) (Palese, 2007). To date, 18 HA and 11 NA subtypes have been reported (Palese, 2007; Tong et al., 2012, 2013). All IAV subtypes (with the exception of H17N10 and H18N11 identified in fruit bats) have been isolated from wild aquatic birds, which are considered the main natural reservoir (Webster et al., 1992).

Based on the World Organization for Animal health (OIE, Office International des Epizooties), avian-origin influenza viruses are classified in low pathogenic (LPAIV) and high pathogenic (HPAIV) avian influenza viruses, depending on the severity of the disease that they induce in poultry (OIE, 2017). LPAIV strains usually produce relatively mild clinical signs in broilers and reduction in egg production in layers, but promote secondary infections usually associated with enhancement of the pathology and outbreaks with increased mortality (Mo et al., 1997; Alexander, 2000). HPAIV strains can be responsible for systemic and fatal infections with high mortality rates in poultry (Mo et al., 1997; Alexander, 2000). Both LPAIV and HPAIV have an enormous economic impact in the poultry industry and they also represent a risk to public health (Shen et al., 2014; Khan et al., 2015). H9N2 IAV strains are classified as LPAIV. The first H9N2 IAV (A/turkey/Wisconsin/1/1966, WI/66) was isolated from an outbreak in turkeys in Wisconsin in 1966 (Homme and Easterday, 1970; Khan et al., 2015). Since the late 1980s and early 1990s, when they were isolated from poultry in Hong Kong and China (Perez et al., 2003a; Li et al., 2005; Sun and Liu, 2015), H9N2 viruses have become endemic in poultry in many parts of Asia, the Middle East, and Africa (Naeem et al., 1999; Nili and Asasi, 2003; Aamir et al., 2007; Lebarbenchon et al., 2008, 2015; Xu et al., 2012; Lee and Song, 2013; Tonnessen et al., 2013; Body et al., 2015; Khan et al., 2015; El Houadfi et al., 2016). H9N2 have been also isolated from humans and pigs in China and Hong Kong (Peiris et al., 1999; Peiris et al., 2001; Perez et al., 2003b; Xu C. et al., 2004; Xu X. et al., 2004; Butt et al., 2005; Yu et al., 2008; Huang et al., 2015; He et al., 2016; Wang et al., 2016; Organization, 2017; Pan et al., 2017; Xu et al., 2017; Yuan et al., 2017). Humans infected with H9N2 IAV usually show mild or no symptoms of illness (Peiris et al., 1999; Butt et al., 2005; Malik Peiris, 2009) and, to date, no human-to-human transmissions have been reported (Uyeki et al., 2002). H9N2 IAV display characteristics of great public health concern: (i) they have been isolated from different mammalian species, including pigs, minks, plateau pikas, and humans (Peiris et al., 1999; Cong et al., 2007; Yu et al., 2008, 2014; Blair et al., 2013; Huang et al., 2015; He et al., 2016; Pan et al., 2017; Xu et al., 2017; Yong-Feng et al., 2017; Yuan et al., 2017); (ii) there is serological evidence of frequent infections in humans (Lu et al., 2008; Kayali et al., 2010; Gray et al., 2011; Pawar et al., 2012; Uyeki et al., 2012; Yang et al., 2012; Coman et al., 2013; Huang et al., 2013; Okoye et al., 2013; Gomaa et al., 2015; Heidari et al., 2016; Li et al., 2016, 2017); (iii) they preferentially bind “human-like” sialic acid receptors (Wan and Perez, 2007); and, (iv) small changes lead to efficient airborne transmission in the ferret animal model and by direct contact in pigs (Wan et al., 2008; Sorrell et al., 2009; Kimble et al., 2011, 2014; Obadan et al., 2015; Li et al., 2017). The internal gene segments of poultry-adapted H9N2 viruses show a remarkable tendency to reassort with other avian-origin IAV that have resulted in the emergence of multiple zoonotic strains of various subtypes, some associated with high mortality, such as the H5N1 and H7N9 viruses, among others (Lin et al., 2000; Park et al., 2011; Monne et al., 2013;Shen et al., 2014; Chaudhry et al., 2015; Naguib et al., 2017; Yong-Feng et al., 2017). The molecular mechanisms that have allowed H9N2 viruses’ ability to replicate in mammals remain largely poorly elucidated.

Host innate immune responses play an important role in the defense against viral infections, including influenza (Iwasaki and Pillai, 2014). The defense is mediated primarily by the induction of an interferon (IFN) response that leads to the expression of an elevated number of IFN-stimulated genes (ISGs), of which some have antiviral activity (Iwasaki and Pillai, 2014). In the case of IAV, the multifunctional non-structural protein 1 (NS1) encoded by segment eight of the viral genome is the main viral factor that antagonizes cellular IFN and ISG responses, allowing viral replication in infected cells (Garcia-Sastre et al., 1998; Talon et al., 2000; Fernandez-Sesma et al., 2006; Newby et al., 2007; Hale et al., 2008). NS1 counteracts innate immune responses by several mechanisms including, among others, the inhibition of IFN production at the pre-transcriptional level through sequestration of double-stranded RNA (dsRNA) and thus decreasing the activation of retinoic acid-inducible gene 1 (RIG-I) (Guo et al., 2007; Mibayashi et al., 2007). NS1 can inhibit the tripartite motif family 25 (TRIM-25)-mediated RIG-I ubiquitination (Gack et al., 2009), and/or the expression of ISGs (Hale et al., 2008), and/or directly inhibit specific ISGs, such as RNase L (Garcia-Sastre, 2001) and protein kinase R (PKR) (Min et al., 2007). In addition, the NS1 protein from some IAV strains blocks pre-mRNA processing and the nuclear export of mRNAs leading to inhibition of host gene expression including that of IFN genes, ISGs and pro-inflammatory cytokines (Fortes et al., 1994). By binding the 30-kDa subunit of the cleavage and polyadenylation specificity factor (CPSF30), NS1 blocks the processing of mRNAs (Nemeroff et al., 1998; Noah et al., 2003; Kochs et al., 2007; Das et al., 2008). Although binding to CPSF30 is not completely essential for IAV, it is possible that inhibition of host gene expression represents an advantage to the virus. For instance, by binding to CPSF30, IAV can inhibit expression of host genes with antiviral activity (e.g., cytokines). Likewise, by inhibiting host gene expression, IAV prevents infection with other pathogens that can compete with viral infection. However, to date, the reasons why the NS1 protein of some IAV and not others interact with CPSF30 are not completely understood, although recent reports suggest that could be related to a mechanism of adaptation to a new host (Chauche et al., 2017; Nogales et al., 2017a). Single as well as different combinations of amino acid residues in NS1 are important for interaction with CPSF30 and, in consequence, inhibition of host gene expression (Kochs et al., 2007; Twu et al., 2007; Hale et al., 2010; Ayllon et al., 2014; DeDiego et al., 2016; Nogales et al., 2017a,c).

In this study, we analyzed the ability to inhibit host gene and innate immune responses of the NS1 protein from A/quail/Hong Kong/G1/1997 (HK/97) H9N2, a prototype strain of the predominant G1-like lineage (Group, 2013). We found that the NS1 protein of HK/97 H9N2 does not have the ability to inhibit host gene expression but efficiently counteracts IFN responses by blocking the induction of IFN and ISGs. We also found that the inability to block host gene expression is a common trait in the NS1 proteins from recent H9N2 IAV strains. Contrary, the NS1 protein from earlier H9N2 IAV strains, including the first H9N2 (WI/66), were able to inhibit both IFN and host gene expression. Importantly, we have been able to identify a new combination of amino acid residues responsible for inhibition of host gene expression and demonstrate that substitution of these amino acid residues in HK/97 NS1 for those present in WI/66 resulted in the recovery of binding to CPSF30 and its ability to inhibit host gene expression. Our results also confirm previous studies demonstrating strain specific differences in the ability of IAV NS1 proteins to inhibit host gene expression.

## Materials and Methods

### Cell Lines

Human embryonic kidney 293T, HEK293T (American Type Culture Collection, ATCC, CRL-11268), human lung epithelial carcinoma A549 (ATCC CCL-185) and UMNSAH/DF-1 chicken embryo fibroblast, DF-1 (ATCC CRL-12203) cells were grown in Dulbecco’s modified Eagle’s medium (DMEM; Mediatech, Inc.) supplemented with 10% fetal bovine serum (FBS) and 1% PSG (penicillin, 100 units/ml; streptomycin 100 μg/ml; L-glutamine, 2 mM) at 37 and 39°C, respectively, with 5% CO_2_.

### Plasmids

The ambisense pDP-based plasmids containing the NS gene from H9N2 strains A/turkey/Wisconsin/1/1966 (WI/66), A/duck/Hong Kong/702/1979 (HK/79), A/quail/Hong Kong/A28945/1988 (HK/88), A/quail/Hong Kong/G1/1997 (HK/97) and A/guinea fowl/Hong Kong/WF10/1999 (HK/99) were generated using standard molecular biology techniques as previously described (Perez et al., 2017). Polymerase II expression pCAGGS plasmids (Niwa et al., 1991) encoding the wild-type (WT) NS1 protein from the H9N2 IAV fused to an HA epitope tag (YPYDVPDYA) at the N terminus (pCAGGS-HA-NH2) (Mibayashi et al., 2007), were generated using standard molecular biology techniques. Briefly, the NS1 open reading frames (ORFs) were amplified by PCR from pDP plasmids using oligonucleotides containing flanking SmaI and XhoI restriction sites for cloning into pCAGGS-HA-NH2 (Mibayashi et al., 2007). WI/66 and HK/97 NS1 ORFs were also cloned into a pGEM-T shuttle plasmid (Promega) to introduce the different mutations by site-directed mutagenesis (Stratagene). After site-directed mutagenesis, NS1 mutants were sub-cloned into the pCAGGS-HA-NH2 vector using SmaI and XhoI restriction sites. To create the different chimeric constructs, KpnI and XbaI restriction sites were introduced at amino acid positions 84 and 139 in the pGEM-T WI/66 NS1 and pGEM-T HK/97 NS1, respectively, using site-directed mutagenesis. To construct the NS1 chimeras HK/97 1-84 and WI/66 1-84, pGEM-T WI/66 NS1 and pGEM-T HK/97 NS1 plasmids were digested with SmaI and KpnI and after DNA purification, inserts were exchanged. For the construction of HK/97 1-139 and WI/66 1-139 chimeras, pGEM-T WI/66 NS1 and pGEM-T HK/97 NS1 plasmids were digested with SmaI and XbaI and inserts were exchanged. Next, NS1 chimeras were subcloned from the pGEM-T into the pCAGGS-HA-NH2 using SmaI and XhoI restriction enzymes. NS1 variants under the control of the phage T7 polymerase were subcloned from pCAGGS-HA-NH2 into pcDNA3 using EcoRI and XhoI restriction enzymes. pCAGGS and pcDNA3 plasmids encoding the NS1 ORFs from influenza A/Puerto Rico/8/34 (PR8) H1N1 and A/Texas/36/1991 (TX) H1N1 viruses were previously described (Kochs et al., 2007; Mibayashi et al., 2007). The pCAGGS plasmid expressing a FLAG-tagged (DYKDDDDK) human CPSF30 has been previously described (Kochs et al., 2007). All plasmid constructs were confirmed by sequencing (ACGT, Inc.) and protein expression was evaluated by Western blot. Primers used for the construction of the different plasmids are available under request.

### Inhibition of Host Gene Expression

To evaluate the effect of NS1 proteins on inhibition of host gene expression, HEK293T or DF-1 cells (24-well plate format, 2.5 × 10^5^cells/well, triplicates) were transiently co-transfected in suspension, using Lipofectamine2000 (Invitrogen), with 1 μg/well of pCAGGS-HA-NH2 NS1 protein expression plasmids, or an empty pCAGGS-HA-NH2 as internal control, together with 25 ng/well of pCAGGS plasmids expressing Gaussia luciferase (Gluc) (Capul and de la Torre, 2008) and the green fluorescent protein (GFP) (Kochs et al., 2007) and placed at 37°C. At 12 h post-transfection (hpt), the medium was replaced and cells were incubated 24 h at 37°C. Subsequently, cells were analyzed for GFP expression and photographed using a fluorescent microscope (Olympus IX81) and camera (QIMAGING, Retiga 2000R). Gluc activity was measured from the tissue culture supernatants (TCS) using a Biolux Gaussia luciferase reagent (New England Bio-Labs) and quantified with a Lumicount luminometer (Packard). The mean values and standard deviations (SDs) were calculated using Microsoft Excel software. Transfected cells were collected and cell lysates were prepared to evaluate levels of protein expression by Western blot assays.

### Inhibition of IFN-β and ISRE Promoter Activation

To evaluate the effect of NS1 proteins on the inhibition of IFN-β and IFN stimulated response element (ISRE) promoters, HEK293T cells (12-well plate format, 3 × 10^5^ cells/well, triplicates) were transiently co-transfected, using a calcium phosphate-based mammalian transfection kit (Stratagene), with 2 μg/well of the pCAGGS-HA-NH2 NS1-expressing plasmids, or empty pCAGGS-HA-NH2 plasmid as control, together with 0.25 μg/well of plasmids expressing Firefly Luciferase (FFluc) under the control of the IFN-β (pIFN-β-FFluc) or the ISRE (pISRE-FFluc) promoters (Kochs et al., 2007). A SV40 Renilla luciferase (Rluc) expression plasmid was included to normalize transfection efficiencies. At 20 hpt, cells were infected (multiplicity of infection, MOI, of 3) with Sendai virus (SeV), Cantell strain (Kochs et al., 2007), and at 18 h post-infection (hpi) FFluc and Rluc activities were quantified from cell lysates using a Dual-Luciferase Reporter Assay System (Promega) and a Lumicount luminometer (PacKard). The mean values and standard deviations (SDs) were calculated using Microsoft Excel software. Transfected cells were recovered and cell lysates were used to evaluate protein expression levels by Western blot.

### Western Blots

Total proteins from transfected HEK293T or DF-1 cell lysates were separated using 10% SDS-polyacrylamide gels and transferred to nitrocellulose membranes. Membranes were blocked for 1 h with 5% dried skim milk in phosphate-buffered saline (PBS) containing 0.1% Tween 20 (PBS-Tween) and incubated overnight at 4°C with rabbit anti-HA (Sigma) or anti-FLAG (Sigma) polyclonal antibodies (pAb) to detect expression of NS1 and CPSF30, respectively. A monoclonal antibody (mAb) specific for actin (Sigma) was used as an internal loading control. To detect the primary antibodies, anti-rabbit (pAb) or anti-mouse (mAb) IgG antibodies conjugated to horseradish peroxidase (HRP; GE Healthcare) were used. Proteins were detected by chemiluminescence using the Super Signal West Femto maximum-sensitivity substrate (Thermo Scientific) following the manufacturer’s recommendations and photographed using a Kodak Image Station.

### Co-immunoprecipitation of NS1 and CPSF30

For production of human CPSF30, HEK293T cells (6-well plate format, 1.5 × 10^6^ cells/well) were transiently transfected with 2 μg/well of a pCAGGS plasmid expressing a FLAG-tagged human CPSF30 (Kochs et al., 2007) or empty plasmid as control. At 48 hpt, cells were lysed in 20 mM Tris-HCl (pH 7.5), 100 mM NaCl, 0.5 mM EDTA, 5% glycerol and 1% TritonX-100, supplemented with a complete Mini protease inhibitor cocktail (Roche). HA tagged-NS1 variants were synthesized *in vitro* from the pcDNA3 plasmids using the TNT7 transcription/translation kit (Promega), following manufacturer’s recommendations. Cleared cell lysates expressing the FLAG-tagged CPSF30 were incubated overnight at 4°C with the *in vitro* synthesized HA-tagged NS1 proteins and 30 μl of an anti-FLAG affinity resin (Sigma-Aldrich). After washing three times in Tris-Buffered Saline (TBS) containing 0.1% Tween-20, precipitated proteins were dissociated from the resin using Laemmli buffer and high temperature (95°C) and analyzed by Western blot as described above using rabbit anti-HA(NS1) and anti-FLAG (CPSF30) specific pAbs (Sigma).

### Immunofluorescence Assay (IFA)

To evaluate the subcellular localization of NS1 proteins, human A549 cells or DF-1 chicken embryo fibroblast (24-well plate format, 10^5^ cells/well) cells were transiently transfected with 1 μg/well of the respective pCAGGS expression plasmids containing an N-terminal HA-tagged NS1 constructs, using lipofectamine 2000 (LPF2000) (Invitrogen). At 24 hpt, cells were fixed with 4% (vol/vol) formaldehyde diluted in 1X PBS, permeabilized with 0.1% (vol/vol) Triton X-100, and blocked using 2.5% bovine serum albumin (BSA) diluted in 1X PBS. After blocking, transfected cells were incubated with a polyclonal antibody (pAb) against the HA epitope tag (Sigma, 1:1,000) and probed with a FITC-conjugated donkey anti-rabbit secondary antibody (Dako). Staining with 4′,6-diamindino-2-phenylindole (DAPI) was used to visualize cell nuclei. Protein expression was observed under a Leica fluorescent microscope. Microscope images were colored using Adobe Photoshop CS4 (v11.0) software.

## Results

### The NS1 Protein From A/Quail/Hong Kong/G1/1997 (HK/97) H9N2 IAV Does Not Block Host Gene Expression in Human or Avian Cells

**FIGURE 1:**
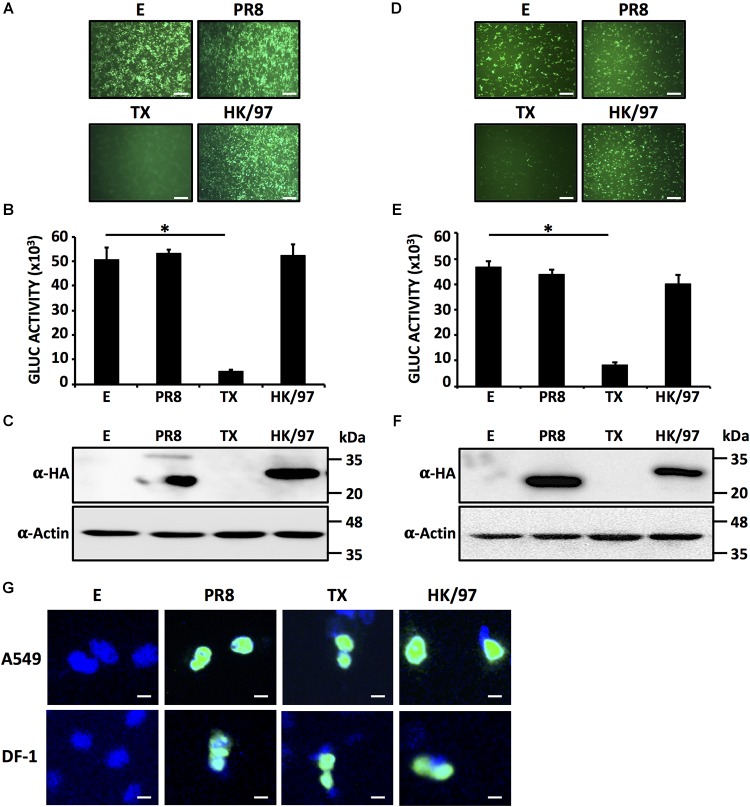
HK/97 H9N2 NS1 protein does not inhibit host gene expression in human HEK293T or avian DF-1 cells: HEK293T **(A–C)** or DF-1 **(D–F)** cells (24-well plate format, 2.5 × 10^5^ cells/well, triplicates) were transiently co-transfected with 1 μg/well of pCAGGS expression plasmids encoding the indicated NS1 proteins fused to an HA epitope tag, or an empty (E) plasmid as a control, together with 25 ng/well of pCAGGS plasmids encoding GFP and Gluc. At 24 hpt, cells were visualized for GFP expression under a fluorescent microscope **(A,D)** and Gluc activity was quantified from TCS **(B,E)**. NS1 protein expression levels from total cell lysates were analyzed by Western blot **(C,F)** using an anti-HA (α-HA) pAb. Actin was included as internal loading control. Molecular mass markers (in kilodaltons, kDa) are indicated. Representative images of three independent transfections are represented. Scale bar, 200 μm **(A,D)**. Results represent the mean and standard deviations (SDs) of triplicate values **(B,E)**. ^∗^*P* ≤ 0.0001 using One-way ANOVA. Molecular protein markers in kDa are indicated on the right of each of the Western blots **(C,F)**. E, empty plasmid; PR8, A/Puerto Rico/8/34 H1N1; TX, A/Texas/36/1991 H1N1; HK/97, A/quail/Hong Kong/G1/1997 H9N2. Subcellular localization of PR8, TX and HK/97 NS1 proteins in transfected human A549 (top) or avian DF-1 (bottom) cells were evaluated at 24 hpt using an anti-HA pAb **(G)**. Nuclei were stained with DAPI. Scale bar, 100 μm.

In order to evaluate if the NS1 protein from A/quail/Hong Kong/G1/1997 (HK/97) H9N2 IAV could block host gene expression, human HEK293T or avian DF-1 cells were co-transfected with expression plasmids encoding GFP and Gluc together with a plasmid encoding the NS1 gene of HK/97 (Figure 1). A plasmid expressing the NS1 protein from A/Puerto Rico/8/34 H1N1 (PR8) was used as a control that fails to block host gene expression, whereas a plasmid expressing the NS1 protein from A/Texas/36/1991 H1N1 (TX) was included as a control that effectively blocks host gene expression (Kochs et al., 2007). Cells transfected with empty (E) plasmid were also included as internal control. At 24 hpt, GFP expression was evaluated using fluorescence microscopy (Figures 1A,D) and Gluc expression levels were quantified in a Luminometer (Figures 1B,E). As expected, PR8 NS1 did not block expression of GFP or Gluc while TX NS1 inhibited the expression of both reporter genes (Kochs et al., 2007). HK/97 NS1 failed to inhibit expression of either GFP or Gluc in human and avian cells. The PR8 and HK/97 NS1 proteins were readily detected by Western blot (Figures 1C,F). In contrast, the TX NS1 protein was not detected as it has been shown previously to inhibit its own synthesis (Kochs et al., 2007). Notably, similar results were obtained in both human (Figures 1A–C) and avian (Figures 1D–F) cells, suggesting that the lack of inhibition of host gene expression by HK/97 NS1 was not related to the host’s cells origin. Furthermore, we have observed the same nuclear subcellular localization for PR8, TX and HK/97 NS1 proteins in both human A549 or avian DF-1 transfected cells (Figure 1G).

### Inhibition of Host Gene Expression by Different H9N2 IAV NS1 Proteins

**FIGURE 2:**
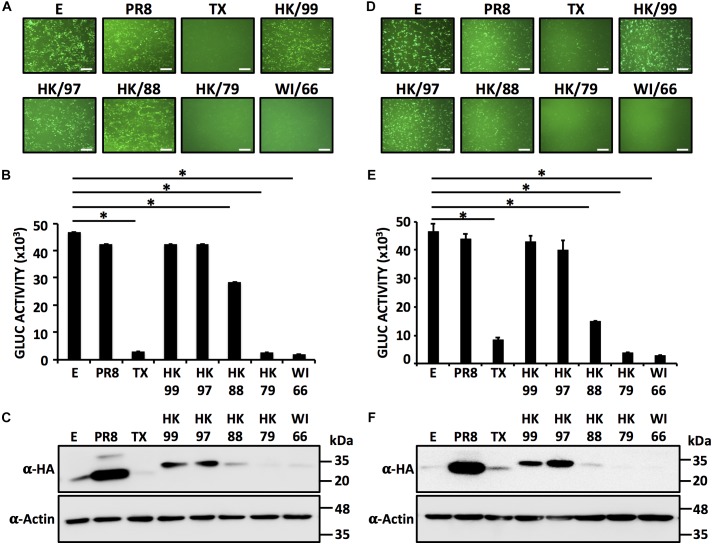
Inhibition of host protein expression by different H9N2 NS1 proteins: HEK293T **(A–C)** or DF-1 **(D–F)** cells (24-well plate format, 2.5 × 10^5^ cells/well, triplicates) were transiently co-transfected with 1 μg/well of pCAGGS plasmids encoding the indicated NS1 proteins fused to an HA epitope tag, or an empty (E) plasmid as internal control, together with 25 ng/well of pCAGGS plasmids encoding GFP and Gluc. At 24 hpt, cells were analyzed for GFP **(A,D)** and Gluc **(B,E)** expression as indicated in Figure 1. ^∗^*P* ≤ 0.0001 using One-way ANOVA. NS1 protein expression levels from total cell lysates were analyzed by Western blot **(C,F)** using an anti-HA (α-HA) pAb. Actin was included as internal loading control. Molecular mass markers (in kDa) are indicated. Representative images of three independent transfections are represented. Scale bar, 200 μm **(A,D)**. Results represent the mean and SDs of triplicate values. Molecular protein markers in kDa are indicated on the right of each of the Western blots **(C,F)**. E, empty plasmid; PR8, A/Puerto Rico/8/34 H1N1; TX, A/Texas/36/1991 H1N1; HK/99, A/guinea fowl/Hong Kong/WF10/1999 H9N2; HK/97, A/quail/Hong Kong/G1/1997 H9N2; HK/88, A/quail/Hong Kong/A28945/1988 H9N2; HK/79, A/duck/Hong Kong/702/1979 H9N2; WI/66, A/turkey/Wisconsin/1/1966 H9N2.

Next, we explored whether the NS1 proteins from different H9N2 strains would share the same phenotype displayed by the NS1 from the HK/97 strain (Figure 2). HEK293T or DF-1 cells were co-transfected with plasmids encoding the NS1 protein from A/turkey/Wisconsin/1/1966 (WI/66), A/duck/Hong Kong/702/1979 (HK/79), A/quail/Hong Kong/A28945/1988 (HK/88) or A/guinea fowl/Hong Kong/WF10/1999 (HK/99) together with expression plasmids encoding GFP and GLuc (Figure 2). Empty plasmid or plasmids encoding the NS1 protein of HK/97, PR8 and TX were included as controls. At 24 hpt, GFP (Figures 2A,D) and Gluc (Figures 2B,E) expression was evaluated. Interestingly, a pattern was realized in which the NS1 proteins from most recent isolates (HK/99 and HK/97) failed to inhibit gene expression [both GFP, Figures 2A,D; and Gluc expression (Figures 2B,E)] whereas those from early isolates (HK/79 and WI/66) effectively inhibited the expression of both reporter genes. Notably, the HK/88 NS1 had an intermediate phenotype, inhibiting only partially the expression of both reporters. Consistent with these observations, the NS1 proteins from HK/99, HK/97, (and PR8) were easily detected by Western blot but not those derived from the HK/79 and WI/66 (and TX) strains (Figures 2C,F). The HK/88 NS1 was detected with a lower intensity than PR8, HK/97 and HK/99 NS1 proteins, consistent with its lower capacity to inhibit gene expression. Notably, results were similar in both cell lines, suggesting that inhibition of host gene expression, or lack thereof, by the NS1 proteins from H9N2 IAV is cell type independent. Importantly, these results indicate that differences exist among the NS1 proteins encoded in H9N2 viruses in their ability to block host gene expression.

### Identification of the Region in H9N2 IAV NS1 Involved in the Ability to Inhibit Host Gene Expression

**FIGURE 3:**
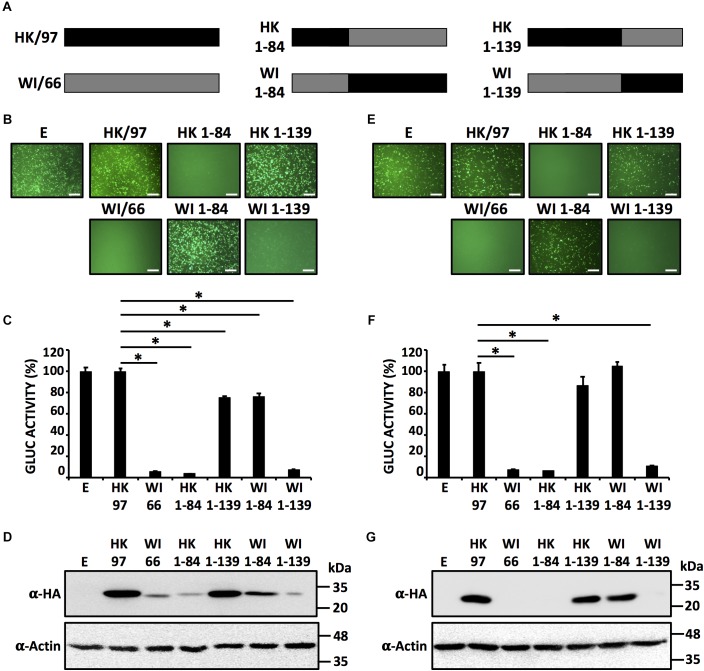
Identification of the region in H9N2 NS1 involves in inhibition of host gene expression. **(A)** Schematic representation of HK/97 (black bars) and WI/66 (gray bars) WT and chimeric (HK 1-84, HK 1-139, WI 1-84 and WI 1-139) NS1 constructs. HEK293T **(B–D)** and DF-1 **(E–G)** cells were transiently co-transfected as described in Figure 1. At 24 hpt, cells were analyzed for GFP **(B,E)** and Gluc **(C,F)** expression as indicated in Figure 1. ^∗^*P* ≤ 0.0001 using One-way ANOVA. NS1 protein expression levels from total cell lysates were analyzed by Western blot **(D,G)** using an anti-HA (α-HA) pAb. Actin expression levels were used as an internal loading control. Molecular mass markers (in kDa) are indicated. Representative images of three independent transfections are represented. Scale bar, 200 μm **(B,E)**. Data were represented as relative activity considering the activity of HK/97 as 100%. Data represent the mean and SDs of triplicate values **(C,F)**. Molecular protein markers in kDa are indicated on the right of each of the Western blots **(D,G)**. E, empty plasmid; A/guinea fowl/Hong Kong/WF10/1999 H9N2; HK/97, A/quail/Hong Kong/G1/1997 H9N2; WI/66, A/turkey/Wisconsin/1/1966 H9N2.

In order to evaluate which region(s) in the NS1 of H9N2 IAV is (are) responsible for influencing host gene expression, we constructed chimeras between the NS1 proteins from HK/97 (unable to inhibit host gene expression) and WI/66 (able to inhibit host gene expression) (Figure 3A). To evaluate the contribution of the N-terminal RNA binding domain (amino acids ∼1–80) and the C-terminal effector region (amino acids ∼81–230) of H9N2 NS1 proteins to inhibition of host gene expression, we introduced a unique KpnI restriction site and generated NS1 chimeric constructs containing the N-terminal domain of one H9N2 NS1 (amino acids 1–84) and the C-terminal domain of the other H9N2 NS1 (amino acids 85–230) fused to an HA N-terminal tag (Figure 3A). Moreover, and since the NS1 region between amino acids 81 and 113 has been previously shown to contain amino acid residues important for inhibition of host gene expression (Kochs et al., 2007), we also introduced a unique XbaI restriction site at position 139 to generate chimeric constructs containing the first N-terminal 139 amino acids of one H9N2 NS1 and the C-terminal 140–230 amino acids of the other H9N2 NS1 with the same N-terminal HA epitope tag (Figure 3A). We tested the ability of both WT and chimeric NS1 proteins to block host gene expression in HEK293T (Figures 3B–D) and DF-1 (Figures 3E–G) cells. As we previously showed (Figures 1, 2), HK/97 NS1 protein did not inhibit reporter gene expression. Likewise, chimera HK 1-139 was unable to inhibit expression of both reporter genes (Figures 3B,C,E,F). In contrast, the NS1 chimeras HK 1-84 and WI 1-139 behave similar to WI/66 and inhibited reporter gene expression (Figures 3B,C,E,F). Importantly, these results were accompanied by the expected pattern of NS1 expression for each of the chimeric constructs (Figures 3D,G). Taken together, these results suggest that a region between amino acids 85 and 139 in H9N2 NS1 is responsible for the control of host gene expression.

### Identification of Amino Acid Residues Involved in the Ability of H9N2 IAV WI/66 NS1 Protein to Inhibit Host Gene Expression

**FIGURE 4:**
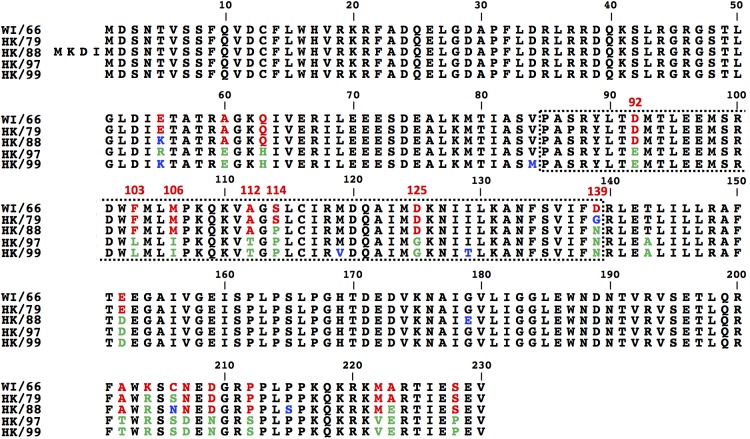
Amino acid sequence alignment of H9N2 NS1 proteins: Doted box indicates the NS1 region involved in the ability to inhibit host gene expression (85–139). In red are indicated the residues from WI/66 NS1 that are different from those present in HK/97 NS1 (green). In blue are indicated the amino acid residues different in other H9N2 NS1 proteins. WI/66, A/turkey/Wisconsin/1/1966 H9N2; HK/79, A/duck/Hong Kong/702/1979 H9N2; HK/88, A/quail/Hong Kong/A28945/1988 H9N2; HK/97, A/quail/Hong Kong/G1/1997 H9N2; HK/99, A/guinea fowl/Hong Kong/WF10/1999 H9N2.

**FIGURE 5:**
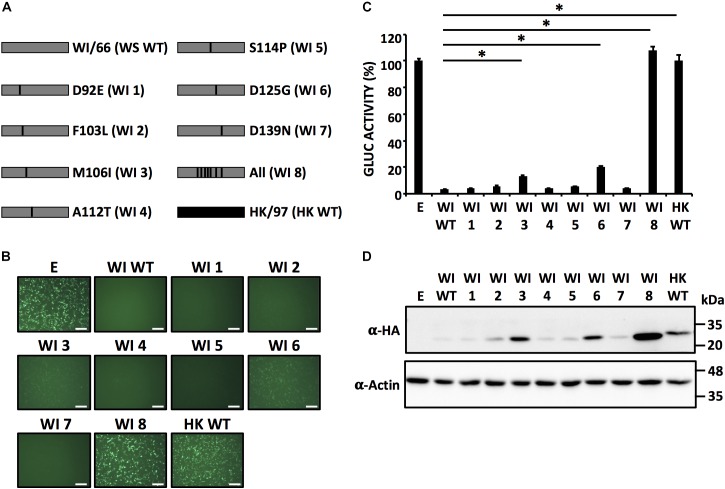
Effect of single amino acid substitutions in WI/66 NS1 on inhibition of host gene expression. **(A)** Schematic representation of WT WI/66 (WI WT, gray) and WT HK/97 (HK WT, black) NS1 proteins and the different WI/66 (WI 1 – WI 8) mutant constructs. **(B–D)** HEK293T cells were transiently co-transfected as described in Figure 1. At 24 hpt, cells were analyzed for GFP **(B)** and Gluc **(C)** expression as indicated in Figure 1. ^∗^*P* ≤ 0.0001 using One-way ANOVA. NS1 protein expression levels from total cell lysates were analyzed by Western blot **(D)** using an anti-HA (α-HA) pAb. Actin was included as internal loading control. Molecular mass markers (in kDa) are indicated. Representative images of three independent transfections are represented. Scale bar, 200 μm **(B)**. Data were represented as relative activity considering the activity of HK/97 as 100%. Data represent the mean and SDs of triplicate values **(C)**. Molecular protein markers in kDa are indicated on the right of each of the Western blots **(D)**.

Based on the results obtained with our chimeric H9N2 NS1 constructs (Figure 3) we made an alignment of the NS1 proteins from these H9N2 IAV (Figure 4), with special interest in the region between amino acids 85 and 139. We identified seven amino acids changes between WI/66 and HK/97 (D92E, F103L, M106I, A112T, S114P, D125G, and D139N) that could be involved in the presence/lack of inhibition of host gene expression between the different H9N2 NS1 proteins (Figure 4, red). To test the individual and combined contribution of these amino acid residues in the inhibition of host protein synthesis, we introduced the amino acid changes D92E, F103L, M106I, A112T, S114P, D125G, and D139N individually or together in WI/66 NS1 (Figure 5A). Next, we analyzed the ability of each mutant to block host gene expression in our reporter-based assay in HEK293T cells (Figures 5B,C). Interestingly, each of the single amino acid mutants behaves like the WI/66 WT strain and were able to inhibit gene expression. Only mutants WI 3 (F103L) and WI 6 (D125G) were less active than the WI/66 WT (Figures 5B,C). However, mutant WI 8 in which all seven amino acids were changed for those present in HK/97 NS1, loss completely the ability to inhibit expression of the reporter genes, just like the HK/97 WT NS1 (Figures 5B,C). Consistent with these results were those obtained when we analyzed the levels of NS1 protein expression (Figure 5D). As described for the NS1 protein from other IAV strains (Nogales et al., 2017a), a combination of amino acids, rather than a single one, is required to influence NS1’s ability to block host gene expression.

**FIGURE 6:**
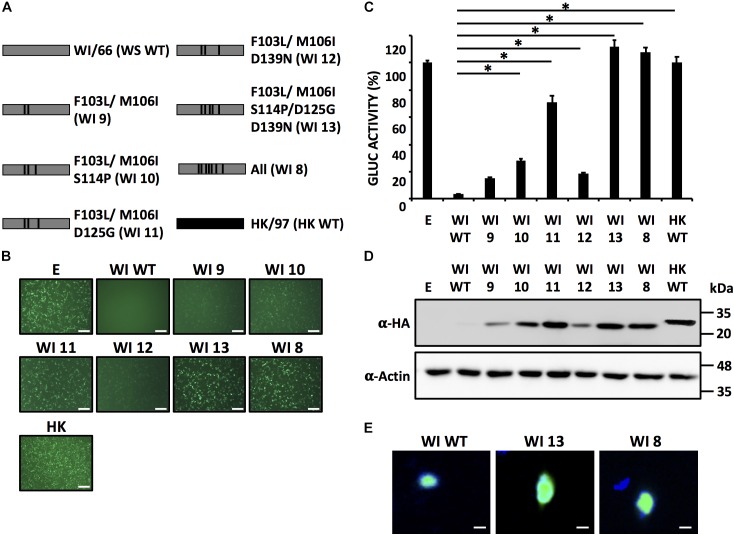
A combination of five amino acid residues (103/106/114/125/139) in WI/66 NS1 is required for inhibition of host gene expression. **(A)** Schematic representation of WT WI/66 (WI WT, gray) and WT HK/97 (HK WT, black) NS1 proteins and the different WI/66 (WI 8 - WI 13) mutant constructs. **(B–D)** HEK293T cells were transiently co-transfected as described in Figure 1. At 24 hpt, cells were analyzed for GFP **(B)** and Gluc **(C)** expression as indicated in Figure 1. NS1 protein expression levels from total cell lysates were analyzed by Western blot **(D)** using an anti-HA (α-HA) pAb. Actin was included as internal loading control. Molecular mass markers (in kDa) are indicated. Representative images of three independent transfections are represented. Scale bar, 200 μm **(B)**. Data were represented as relative activity considering the activity of HK/97 as 100%. Data represent the mean and SDs of triplicate values **(C)**.^∗^*P* ≤ 0.0001 using One-way ANOVA. Molecular protein markers in kDa are indicated on the right of each of the Western blots **(D)**. **(E)** Subcellular localization of WI WT (left), WI 13 (middle) and WI 8 (right) NS1 proteins in transfected human A549 cells were evaluated at 24 hpt using an anti-HA pAb. Nuclei were stained with DAPI. Scale bar, 100 μm.

Our results with individual mutants (Figure 5) suggested that a combination of more than one amino acid in WI/66 H9N2 NS1 protein is necessary for the loss of inhibition of host gene expression. In the case of PR8 NS1 protein, amino acid changes S103F and I106M restored the ability of PR8 NS1 binding to CPSF30 and concomitant inhibition of host gene expression (Kochs et al., 2007). The WI/66 NS1 protein has the same amino acids F103 and M106 than PR8 NS1, but when we exchanged them individually, only minimal effects were observed (Figure 5). Thus, we produced the F103L and M106I WI/66 NS1 double mutant and use it as a template to make the rest of amino acid mutant combinations (Figure 6A). The amino acid change D125G was also included because when this single change was introduced (WI 6 mutant) we saw an effect on reporter gene expression (Figure 5). Moreover, the 125 amino acid residue plays a role in the inhibition of host gene expression by the influenza A/California/04/09 (H1N1) strain (Hale et al., 2010; Clark et al., 2017). The amino acid substitutions S114P and D139N were also included since they are located in the region responsible for the loss/gain of inhibition of host gene expression (Figure 3) and are different between the NS1 protein from WI/66 and HK/97 NS1 (Figure 4).

We then evaluated the ability of each combination of amino acids in WI/66 NS1 (Figure 6A) to block host gene expression (Figures 6B,C). Amino acid changes MF103L/M106I (WI 9) had a minimal effect on inhibition of reporter gene expression as compared to WI/66 WT or M106I (WI 3) mutant (Figures 5B,C) indicating that amino acid substitutions F103L and M106I have a minimal effect on the ability of WI/66 NS1 protein to inhibit host gene expression (Figures 6B,C). The triple mutants WI 10 (F103L/M106I/S114P) and WI 12 (F103L/M106I/D139N) were slightly affected, as compared to WI/66 NS1 WT in inhibiting reporter gene expression (Figures 6B,C) while the triple mutant WI 11 (F103L/M106I/D125G) was severely affected in inhibiting host gene expression, and only differed in ∼20% inhibition with HK WT NS1 (Figures 6B,C). Notably, mutant WI 13 (F103L/M106I/S114P/D125G/D139N) was unable to inhibit reporter gene expression (Figures 6B,C), similar to WI 8, that contains the seven amino acid changes, and HK WT NS1 (Figures 6B,C). All the WI NS1 mutants were detected by Western blot (Figure 6D) but with different expression levels, as expected based on the results from reporter gene expression (Figures 6B,C). WI 9, 10, and 12 were detected to lower extend that WI 11 and more notably, WI 13 (Figure 6D). Notably, we observed the same nuclear subcellular localization of NS1 WI WT and WI 8 and WI 13 mutants in transfected human A549 cells (Figure 6E). Altogether, these results indicate that the combination of amino acid residues F103L/M106I/S114P/D125G/D139N in WI/66 NS1 protein is sufficient to loss its ability to inhibit host gene expression.

### Substitutions L103F/I106M/P114S/G125D/N139D Restore the Ability to Inhibit Host Gene Expression in HK/97 IAV NS1 Protein

**FIGURE 7:**
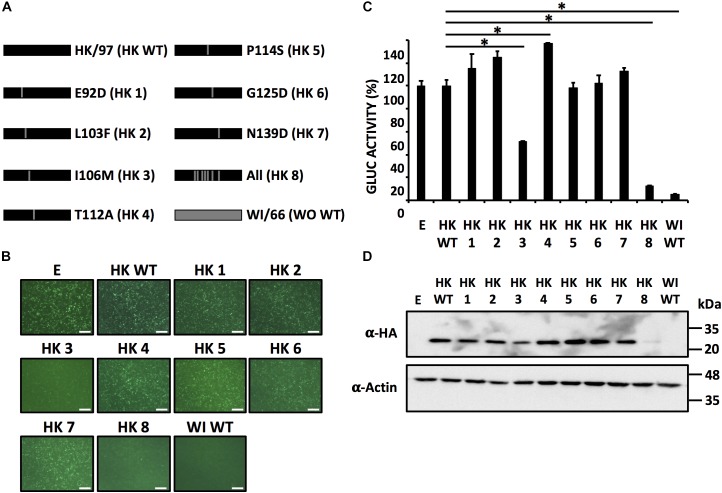
Single amino acid substitutions at positions 92, 103, 106, 112, 114, 125, and 139 do not restore the ability of HK/97 NS1 to inhibit host gene expression. **(A)** Schematic representation of WT NS1 HK/97 (HK WT) (black bars) and WI/66 (WI WT) (gray bar) and the different HK/97 mutants (HK 1 - HK 8) with the amino acid substitutions indicated. **(B–D)** HEK293T cells were transiently co-transfected as described in Figure 1. At 24 hpt, cells were analyzed for GFP **(B)** and Gluc **(C)** expression. NS1 protein expression levels from total cell lysates were analyzed by Western blot **(D)** using an anti-HA pAb (α-HA). Actin was included as internal loading control. Molecular mass markers (kDa) are indicated. **(B)** Representative images of three independent transfections are represented. Scale bar, 200 μm. **(C)** Data represent the mean and SDs of triplicate values. Data were represented as relative activity considering the activity of HK/97 as 100%. ^∗^*P* ≤ 0.0001 using One-way ANOVA. Molecular protein markers in kDa are indicated on the right of each of the Western blots **(D)**.

**FIGURE 8:**
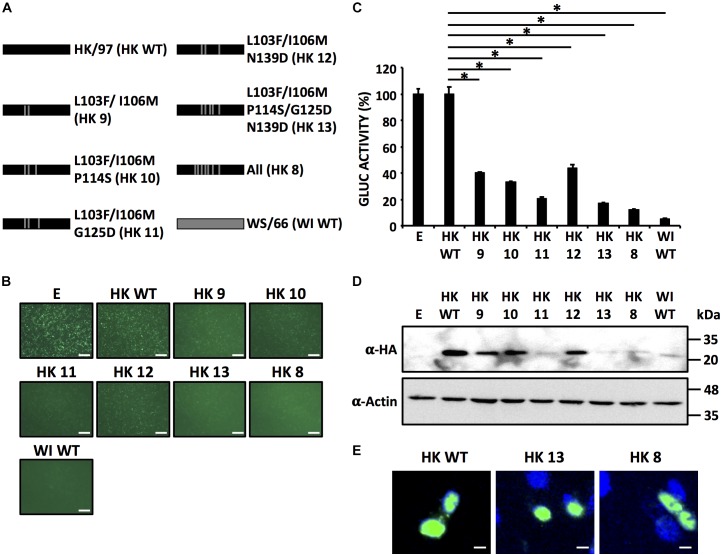
Five amino acid changes (L103F, I106IM, P114S, G125D, and N139D) in HK/97 NS1 restores its ability to block host gene expression. **(A)** Schematic representation of WT NS1 proteins HK/97 (HK WT) (black bars) and WI/66 (WI WT) (gray bar) and the different HK/97 mutants (HK 8 - HK 13) with the amino acid substitutions indicated. **(B–D)** HEK293T cells were transiently co-transfected as described in Figure 1. At 24 hpt, cells were analyzed for GFP **(B)** and Gluc **(C)** expression. NS1 protein expression levels from total cell lysates were analyzed by Western blot **(D)** using an anti-HA pAb (α-HA). Actin was included as internal loading control. Molecular mass markers (in kDa) are indicated. **(B)** Representative images of three independent transfections are represented. Scale bar, 200 μm. **(C)** Data represent the mean and SDs of triplicate values. Data were represented as relative activity considering the activity of HK/97 as 100%. ^∗^*P* ≤ 0.0001 using One-way ANOVA. Molecular protein markers in kDa are indicated on the right of each of the Western blots **(D)**. The subcellular localization of HK WT (left), HK 13 (middle) and HK 8 (right) in human A549 transfected cells were evaluated at 24 hpt by IFA using an anti-HA pAb **(E)**. Nuclei were stained with DAPI. Scale bar, 100 μm.

To assess whether the same residues responsible for the loss of inhibition of host gene expression in WI/66 NS1 could restore the ability of HK/97 NS1 to inhibit host gene expression, we introduce individually the same amino acid changes in HK/97 NS1 (Figure 7A) and evaluated their ability to inhibit host gene expression (Figures 7B,C) as well as NS1 protein expression levels by Western blot (Figure 7D). As expected, none of the single HK/97 NS1 mutants completely restored the ability to inhibit host gene expression (Figures 7B,C) with mutant HK 3 (I106M) showing approximately 50% of inhibition in reporter gene expression as compared to HK/97 WT NS1. Mutant HK 8 containing the seven amino acid changes (E92D/L103F/I106M/T112A/P114S/G125D/N139D) inhibited reporter gene expression to levels comparable to WI/66 WT NS1 (Figures 7B,C), further demonstrating that a combination of more than one amino acid is involved in the ability to inhibit host gene expression. Based on these results, we constructed HK/97 NS1 mutants (Figure 8A) with the same combination of amino acid changes to those described for WI/66 NS1 (Figure 6A). Mutant HK 13 containing five amino acid (L103F/I106M/P114S/G125D/N139D) was able to inhibit reporter gene expression to the same level of WI/66 NS1 WT (Figures 8B,C). The other HK/97 NS1 mutants containing the same combination of amino acid changes than those previously described for WI/66 NS1 showed an intermediate phenotype between the double mutant HK 9 (L103F/I106M) and mutant HK 13 (Figures 8B,C), as previously described for WI/66NS1 (Figures 6B,C). Likewise, expression levels of all NS1 HK/97 mutants was detected by Western blot (Figure 8D) with the exception of HK 11 (L103F/I106M/G125D), HK 13 (L103F/I106M/P114S/G125D/N139D) and HK 8 (E92D/L103F/I106M/T112A/P114S/G125D/N139D) (Figure 8D). As previously shown for WI/66 NS1, amino acid changes in HK/97 NS1 did not affect the subcellular localization in transfected human A549 cells (Figure 8E). These results indicate that amino acid changes in the NS1 of HK/97 at positions 103, 106, 114, 125, and 139 for those in WI/66 NS1 restores the ability to inhibit host gene expression.

### Amino Acid Changes L103F/I106M/P114S/G125D/N139D Restore the Ability of HK/97 IAV NS1 to Bind to CPSF30

**FIGURE 9:**
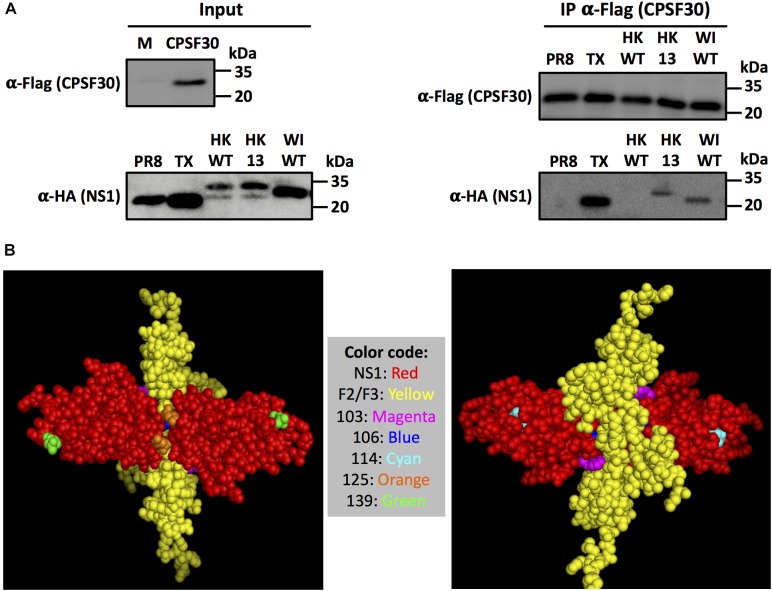
Amino acid changes L103F, I106IM, P114S, G125D and N139D in H9N2 NS1 are required for interaction with CPSF30. **(A)** Analysis of NS1-CPFS30 interaction by co-immunoprecipitation. CFSF30 was expressed in HEK293T cells, mixed with the indicated NS1 proteins synthetized *in vitro* and immunoprecipited using an anti-FLAG resin. Input (left) and immunoprecipitated (right) proteins were detected by Western blot using antibodies specific against the FLAG (CPSF30) or HA (NS1) epitope tags. Molecular mass markers (in kDa) are indicated. **(B)** Tridimensional structure of the NS1 effector domain binding to F2/F3 region of CPSF30: Monomers of IAV NS1 protein are represented in red. The F2/F3 region in CPSF30 involved in interaction with NS1 is represented in yellow. NS1 residues 103, 106, 114, 125, and 139 involved in interaction with CPSF30 are showed in different colors in the figure legend. The model structure was generated using Cn3D and is based on the NS1 of A/Udorn/72 H3N2 (PDB entry 2RHK).

We and others have previously described that NS1 proteins from some IAV strains interact with the cellular host factor CPSF30 and this interaction is responsible for the NS1’s ability to inhibit host gene expression (Nemeroff et al., 1998; Kochs et al., 2007; Ayllon et al., 2014; DeDiego et al., 2016; Clark et al., 2017; Nogales et al., 2017a,b). To evaluate if the five amino acid substitutions L103F/I106M/P114S/G125D/N139D in HK/97 NS1 restore its ability to bind to CPSF30, cell extracts from HEK293T transfected with a pCAGGS plasmid expressing a FLAG-tagged CPSF30 were incubated with *in vitro* transcribed and translated HK/97 NS1 WT, HK/97 NS1 13 (L103F/I106M/P114S/G125D/N139D) and WI/66 NS1 WT fused to an HA-tag (Figure 9A) and agarose beds conjugated with an anti-FLAG pAb. NS1 proteins from PR8 and TX were included as negative and positive controls, respectively (Kochs et al., 2007). All IAV NS1 proteins were expressed efficiently *in vitro* (Figure 9A, left panel, input). After co-immunoprecipitation, CPSF30 protein was detected to similar levels by Western blot in all cases (Figure 9A, right panel, α-FLAG). As expected, PR8 NS1 failed to interact with CPSF30 while TX NS1 efficiently interacted with CPSF30 (Figure 9A, right panel, α-HA). HK/97 WT NS1 was not detected in the co-immunoprecipitation, suggesting the lack of interaction with CPSF30. However, mutant HK 13 (L103F/I106M/P114S/G125D/N139D) and WI/66 NS1 were detected by Western blot in the co-immunoprecipitation, suggesting binding to CPSF30 (Figure 9A, IP α-HA). These results demonstrate that amino acid substitutions L103F/I106M/P114S/G125D/N139D restored the ability of HK/97 NS1 protein to interact with CPFS30, providing a mechanism for inhibition of host gene expression.

A three-dimensional structure of influenza A/Udorn/72 H3N2 NS1 (red) (Das et al., 2008) bound to the F2/F3 domain of CPSF30 (yellow) (Figure 9B) shows that amino acid residues 103 (magenta), 106 (blue) and 125 (orange) are closely located to the NS1-CPSF30 interaction interphase, providing an explanation for the contribution of these amino acid residues in the binding to CPSF30 (Das et al., 2008). NS1 amino acid residues 114 (cyan) and 139 (green) are not in direct contact with CPSF30 (Figure 9B) but could be important for maintaining the quaternary structure of NS1 for its interaction with CPSF30, in combination with residues 103, 106, and 125.

### Inhibition of IFN Responses by IAV H9N2 NS1 Proteins

**FIGURE 10:**
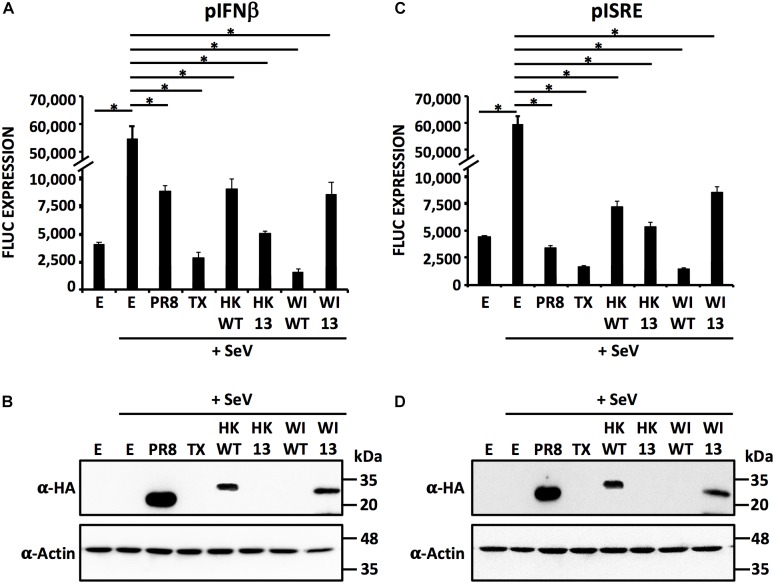
The NS1 protein fromH9N2 IAV counteract IFN responses induced by SeV infection: HEK293T cells (12-well plate format, 3 × 10^5^ cells/well, triplicates) were transient co-transfected, using CaPO_4_, with 2 μg/well of pCAGGS expression plasmids encoding the indicated NS1 proteins fused to an HA epitope tag or an empty (E) plasmid as control, together with 0.25 μg/well of plasmids expressing FFluc under the control of the IFN-β or the ISRE promoters. At 20 hpt, cells were infected with SeV, Cantell strain, and at 18 hpi, cells were analyzed for IFN-β **(A)** and ISRE **(C)** promoter activation by FFluc activity. Results represent the mean and SDs of triplicate values. ^∗^*P* ≤ 0.0001 using One-way ANOVA. **(B,D)** H9N2 NS1 protein expression levels from total cell lysates were analyzed by Western blot using an anti-HA pAb (α-HA). Actin was included as internal loading control. Molecular mass markers (in kDa) are indicated on the right of each of the Western blots.

In order to evaluate the ability of HK/97and WI/66 NS1 proteins and their respective HK 13 (L103F/I106M/P114S/G125D/N139D) and WI 13 (F103L/M106I/S114P/D125G/D139N) mutants to counteract IFN and ISG responses, HEK293T cells were co-transfected with pCAGGS plasmids expressing HK/97 WT, HK 13, WI/66 WT and WI 13 NS1 proteins together with reporter plasmids expressing Firefly luciferase (FFluc) under the control of the IFN-β (Figure 10A) or ISRE (Figure 10C) promoters. Empty (E) plasmid and plasmids expressing PR8 and TX NS1 proteins were included as internal controls. At 18 hpt, cells were mock-infected or infected with SeV for 18 h to induce activation of both promoters. Activation of IFN-β and ISRE promoters was determined by measuring reporter expression levels (Figures 10A,C, respectively). As expected, in cells transfected with E plasmid, SeV infection induced robust activation of the IFN-β (Figure 10A) and ISRE (Figure 10C) promoters while in cells transfected with the different NS1-expressing plasmids, SeV infection induced lower activation of the IFN-β (Figure 10A) and ISRE (Figure 10C) promoters. As expected, and similar to our previous studies (Kochs et al., 2007; Steidle et al., 2010; DeDiego et al., 2016; Chauche et al., 2017; Clark et al., 2017; Nogales et al., 2017a,c,d), inhibition of IFN-β and ISRE promoter activation was more evident with NS1 proteins that were able to inhibit host gene expression (TX, HK 13 and WI WT) than those lacking the ability to inhibit host gene expression (PR8, HK WT and WI 13). When we evaluated NS1 protein expression by Western blot, only the NS1 proteins from PR8, HK WT and WI 13 where detected (Figures 10B,D). These results indicate that amino acid residues103/106/114/125/139 are required for efficient inhibition of host gene expression in H9N2 NS1 proteins but not for inhibition of SeV-mediated activation of IFN-β and ISRE promoters. Moreover, and similar to our previous studies (Kochs et al., 2007; Steidle et al., 2010; DeDiego et al., 2016; Chauche et al., 2017; Clark et al., 2017; Nogales et al., 2017a,c,d), these results demonstrate that the ability of NS1 proteins, including H9N2 IAV, to inhibit host gene expression is independent but help with, their ability to antagonize IFN responses. This can be attributed to a mechanism of host adaptation of some IAV, where inhibition of host protein expression, not only inhibition of IFN-β and/or ISRE, confers an advantage to the replication of the virus (Chauche et al., 2017; Nogales et al., 2017a).

## Discussion

H9N2 IAV have been detected in domestic and wild avian species around the world (Naeem et al., 1999; Nili and Asasi, 2003; Li et al., 2005; Aamir et al., 2007; Xu et al., 2012; Lee and Song, 2013; Tonnessen et al., 2013; Body et al., 2015; Lebarbenchon et al., 2015; El Houadfi et al., 2016). H9N2 IAV have also been isolated from humans (Peiris et al., 1999; Butt et al., 2005; Huang et al., 2015; He et al., 2016; Pan et al., 2017; Xu et al., 2017; Yuan et al., 2017) and pigs (Peiris et al., 2001; Xu X. et al., 2004; Yu et al., 2008; Wang et al., 2016). In humans, the elevated positive cases obtained by the examination of serological samples suggest that H9N2 IAV induce mild or asymptomatic infections that remained undetected (Khan et al., 2015). Since pigs can be infected by avian, human and swine IAV, the concern about a possible reassortment between H9N2s with other strains represents a major public health concern.

The ability of NS1 protein to counteract host innate immune responses and to allow viral replication in IFN-competent organism is common for IAV (Hale et al., 2008). One mechanism of inhibiting IFN responses is mediated by the ability of NS1 proteins from some IAV to interact with the cellular host factor CPSF30, which results in the block of host gene expression, including antiviral and pro-inflammatory cytokine genes (Nemeroff et al., 1998; Noah et al., 2003; Kochs et al., 2007; Das et al., 2008). However, the ability of IAV NS1 proteins to bind to CPSF30 is not universally present among IAV (Nemeroff et al., 1998; Noah et al., 2003; Kochs et al., 2007; Das et al., 2008; Hale et al., 2010; Ayllon et al., 2014; DeDiego et al., 2016; Nogales et al., 2017a,c). To date, the reason why some IAV strains and not others encode NS1 proteins with the ability to inhibit host gene expression remains unknown. However, mounting evidences, including this manuscript, suggest that the lack of inhibition of host gene expression by IAV NS1 proteins represent a mechanism of host adaptation to mammal hosts (Ayllon et al., 2014; DeDiego et al., 2016; Chauche et al., 2017; Clark et al., 2017; Nogales et al., 2017a,c).

In this study, we evaluated the ability of the NS1 protein from different H9N2 IAV isolates to inhibit host gene expression. We showed that HK/97 H9N2 NS1 protein, a reference virus for the G-1 lineage and one of the lineages more distributed in Asia (Group, 2013), is unable to inhibit host gene expression in human or avian cells (Figures 1–3). Contrary, the NS1 protein from the first H9N2 virus isolated, WI/66 (Homme and Easterday, 1970), is able to efficiently inhibit host gene expression in both human and avian cells (Figures 2, 3). The construction of chimeras between HK/97 and WI/66 NS1 proteins mapped the region involved in the lack/presence of inhibition of host gene expression between amino acids 85–139 (Figure 3). Previous studies have identified that residues centered around residue 186 in the canonical CPSF30 binding domain of IAV NS1 (Noah et al., 2003; Twu et al., 2006; Chauche et al., 2017; Nogales et al., 2017a) as well as other newly identified residues outside the classical CPSF30 binding domain in influenza PR8 (residues 103 and 108) (Kochs et al., 2007), pH1N1 (residues 108, 125, and 189 or 55, 90, 123, 125, 131, and 205) (Hale et al., 2010; Clark et al., 2017), circulating human H3N2 (residues 64, 86, 189, and 194) (DeDiego et al., 2016; Nogales et al., 2017c) as well as H7N9 (residue 106) (Ayllon et al., 2014) are important for binding to CPSF30 and inhibition of host gene expression. Analysis of the amino acid sequences in this NS1 region (Figure 4) and the subsequently construction of individual and combined amino acid mutants in WI/66 (Figures 5, 6) and HK/97 (Figures 7, 8) NS1, allowed us to identify a new combination of five amino acid residues (103, 106, 114, 125, and 139) involved in binding to CPSF30 and inhibition of host gene expression in the NS1 protein of H9N2 viruses. Residue changes L103F, I106M, P114S, G125D, and N139D in HK/97 NS1 protein (HK 13 mutant) completely restored the ability of NS1 to bind to CPSF30 (Figure 9). Notably, residues 103, 106, and 125 are located in the NS1 region involve in interaction with CPSF30 (Figure 9) while residues 114 and 139 are not in direct contact with CPSF30 (Figure 9) but could be important for maintaining the proper NS1 structure for interaction with CPSF30. Nevertheless, since we used the crystal structure of A/Udorn/72 H3N2 NS1, it is possible that the identified amino acid residues in H9N2 NS1 protein may display alternative structural features than those shown in the crystal structure of A/Udorn/72 H3N2 NS1.

The NS1 protein from canine (CIV) (Nogales et al., 2017a) and equine (EIV) (Chauche et al., 2017) H3N8 IAV, that have an avian origin, have evolved and acquired mutations that resulted in loss of CPSF30 binding and in consequence lack the ability to inhibit host gene expression (Chauche et al., 2017; Nogales et al., 2017a). It is tempting to speculate that the ability to block host gene expression by avian-origin IAV could be important during the initial stages of IAV adaptation to mammals, however, such feature is lost during adaptation to mammals (Chauche et al., 2017; Nogales et al., 2017a). Alternatively, as avian-origin IAV transition and adapt from replication in the gastrointestinal of wild aquatic birds (anseriformes and charadriiformes) to the respiratory tract of poultry (galliformes) and mammals, NS1’ host gene expression inhibition ability is lost. The fact that NS1 loses control of host gene expression is perhaps another feature that allows avian-origin IAV to jump to mammals. This is consistent with our results in which the NS1 proteins from early H9N2 strains (WI/66 and HK/79) blocked host gene expression while the NS1 proteins from more recent H9N2 IAV (HK/97 and HK/99) did not have this ability. Furthermore, we have also observed an intermediate phenotype with the NS1 protein from HK/88, further supporting this hypothesis. These findings suggest that the ability to inhibit host gene expression by H9N2 NS1 proteins has been lost along viral evolution. Notably, the first H9N2 IAV isolated from humans was in 1999 (Peiris et al., 1999) and from pigs between 1998 and 2000 (Peiris et al., 2001), dates that match with the isolation of the H9N2 IAV whose NS1 proteins did not inhibit efficiently host gene expression. It is worth mentioning that the recent pandemic A/California/04/09 H1N1 (pH1N1) strain was also unable to inhibit host gene expression (Hale et al., 2010) and the residues involved in this lack of inhibition of host gene expression were mapped at amino acids 108, 125, and 189. However, more recent pH1N1 isolates from the last 2015/2016 influenza seasons carry 6 amino acid changes that restored the ability of the NS1 protein to inhibit host gene expression (Clark et al., 2017). Such observation suggests that although the ability to inhibit host gene expression might confer a mechanism of host adaptation for some IAV like the H3N8 CIV (Nogales et al., 2017a), EIV (Chauche et al., 2017) and H9N2 IAV (this study), this situation might be different for other subtypes, e.g., the pH1N1 (Hale et al., 2010; Clark et al., 2017).

In summary, our study contributes in the current knowledge of the mechanism used for IAV to counteract host gene expression and identified a new combination of amino acids required for NS1 binding to CPSF30 and inhibition of host gene expression. Likewise, our studies further demonstrate the presence of strain specific differences in the ability of IAV NS1 proteins to inhibit host gene expression. Moreover, our results also confirm that inhibition of host gene expression by IAV NS1 is important for adaptation of IAV into mammals. Future *in vivo* studies in birds as well as in mammals with H9N2 IAV encoding NS1 proteins that lack/present the ability to counteract host gene expression will help to elucidate their role in viral pathogenicity, transmission and host adaptation.

## Author Contributions

LR, AN, and LM-S conceived and planned the experiments, contributed to the interpretation of the results, and wrote the manuscript. LR and AN carried out the experiments. All authors provided critical feedback and helped shape the research, analysis, and manuscript.

## Conflict of Interest Statement

The authors declare that the research was conducted in the absence of any commercial or financial relationships that could be construed as a potential conflict of interest.
